# Suppressive Activity of Boiogito, a Japanese Traditional Kampo Medicine, on Periostin Secretion in Human Fibroblast-Like Synoviocytes In Vitro

**DOI:** 10.7759/cureus.57690

**Published:** 2024-04-05

**Authors:** Midori Mochizuki, Takayuki Okumo, Haruka Takemura, Kanako Izukashi, Tokito Tatsuo, Hideshi Ikemoto, Naoki Adachi, Nobuyuki Kawate, Masataka Sunagawa

**Affiliations:** 1 Department of Physiology, Showa University Graduate School of Medicine, Tokyo, JPN; 2 Department of Rehabilitation Medicine, Showa University School of Medicine, Tokyo, JPN; 3 Department of Orthopedic Surgery, Showa University Fujigaoka Hospital, Yokohama, JPN; 4 Department of Pharmacology, Showa University Graduate School of Medicine, Tokyo, JPN

**Keywords:** periostin, stat6, interleukin-13, kampo medicine (japanese herbal medicine), osteoarthritis (oa), synoviocytes

## Abstract

Background

Knee osteoarthritis (KOA) is a prevalent degenerative disease that affects the knee joints, particularly among individuals aged over 40 years. It leads to pain, stiffness, and reduced quality of life; affects approximately 300 million individuals worldwide; and is increasing, particularly in developed nations. Although treatments for KOA range from conservative measures to surgical interventions, such as total knee arthroplasty (TKA), the financial burden of TKA in many countries underscores the urgent need for effective conservative therapies. The pathophysiology of KOA involves articular cartilage degeneration, increased subchondral bone turnover, synovitis, and periarticular soft tissue contracture. Abnormal bone turnover, intensified by factors, such as weight gain and knee injury, precedes cartilage degeneration. Synovitis, characterized by inflammation in the synovial tissue, plays a crucial role in perpetuating the disease by triggering a cascade of catabolic and proinflammatory mediators, including cytokines, such as interleukin (IL)-1 beta, tumor necrosis factor-alpha, and IL-13. Periostin, an extracellular matrix protein, is implicated in KOA progression, with its levels increasing with disease severity.

Materials & methods

In this study, the preventive effect of boiogito (BOT), a traditional herbal medicine, on periostin secretion in human fibroblast-like synoviocytes (hFLS) stimulated by IL-13 was investigated. Synoviocyte Growth Medium and recombinant human IL-13 were used for cell culture and stimulation. BOT was dissolved in phosphate-buffered saline and applied to cell cultures. Periostin secretion and mRNA expression were measured using enzyme-linked immunosorbent assay and quantitative reverse transcription polymerase chain reaction, respectively. Cell viability was assessed using an MTT assay, and signal transducer and activator of transcription factor 6 (STAT6) phosphorylation was examined using Western blotting.

Results

IL-13 stimulation of hFLS significantly increased periostin secretion, with levels rising above 20 ng/mL after 72 h of stimulation. Pretreatment with BOT dose-dependently suppressed periostin secretion, with doses of 1,000 μg/mL significantly reducing periostin levels. Furthermore, BOT inhibited periostin mRNA expression and STAT6 phosphorylation in IL-13-stimulated hFLS, suggesting its potential in modulating IL-13-mediated inflammatory pathways in KOA.

Conclusion

This study demonstrated the preventive effect of BOT on periostin secretion in IL-13-stimulated hFLS, highlighting its potential as a therapeutic agent for KOA. By inhibiting periostin production and downstream signaling pathways, BOT may offer a promising conservative treatment option for KOA, addressing the inflammatory cascade implicated in disease progression. Further research is warranted to elucidate the specific herbal components responsible for the therapeutic effects of BOT and to validate its efficacy in clinical settings.

## Introduction

Knee osteoarthritis (KOA) is a degenerative disease that affects the knee joints, which is more common in individuals aged over 40 years and causes pain, stiffness, and impairment of quality of life (QOL) [1]. Approximately 300 million individuals are affected by KOA worldwide. The prevalence of KOA continues to increase, particularly in most developed countries [2]. Therapeutic strategies for KOA can be divided into conservative and surgical treatments [1]. Conservative treatments, encompassing activities, such as locomotor exercise, medication, and orthotics, aim to alleviate pain and relieve functional disabilities associated with KOA [3]. In some cases, where conservative treatment fails to improve impaired QOL, surgical interventions, such as total knee arthroplasty (TKA) or around-knee osteotomy, may become necessary [4]. Notably, TKA poses a financial burden, contributing to 1%-5% of gross domestic products in various countries [5]. Consequently, there is a pressing need to develop effective and preventive conservative treatment options.

The pathophysiology of KOA is characterized by articular cartilage degeneration, increased turnover of subchondral bone, osteophyte formation, synovitis, and contracture of periarticular soft tissues [6]. Abnormal bone turnover, including osteoclast differentiation and proliferation in the subchondral bone, occurs before the development of cartilage degeneration in the initial stage of KOA [7]. This is primarily attributed to increased loading resulting from weight gain, knee injury, and continuous load bearing, among other factors. Secondary factors contributing to the progression of KOA include exacerbation of synovitis, where inflammatory cytokines released by immune cells in the synovium and chemical mediators, such as nitric oxide, trigger a reaction that promotes articular cartilage degeneration and subchondral bone resorption [8]. Synovitis is the inflammation of the synovial tissue. The breakdown products of cartilage released into the synovial fluid are phagocytosed by synovial cells, intensifying synovial inflammation [9]. Consequently, activated synovial cells produce catabolic and proinflammatory mediators, leading to the excessive production of proteolytic enzymes responsible for cartilage degradation in chondrocytes. In addition to its effects on cartilage inflammation and degradation, the inflamed synovium contributes to osteophyte formation via the synovial perlecan reaction [10].

Periostin, which is also known as osteoblast-specific factor 2, is an extracellular matrix protein initially identified in a mouse osteoblast cell line [11]. It is associated with the pathophysiology of arthritis, tumor development, atherosclerosis, and inflammatory diseases [12]. Recent findings suggest that periostin deposition intensifies chronic allergic inflammation [13]. Periostin is considered a crucial structural mediator that contributes to appropriate tissue adaptation in response to injury and KOA progression [14]. This means that elevated periostin levels in joint fluid may promote secondarily the secretion of inflammatory cytokines and cartilage matrix catabolic enzymes, leading to the progression of KOA.

Interleukin (IL)-13 is an anti-inflammatory cytokine that shares receptors with IL-4. A previous study showed that IL-13 administration to human fibroblast-like synoviocytes (hFLS) increased periostin production. Other reports have shown that periostin and IL-13 levels increase with KOA stage progression [15] and that periostin secretion is upregulated in OA synoviocytes via IL-13 stimulation through phosphorylation of the transcription factor signal transducer and activator of transcription factor 6 (STAT6) [16]. Periostin secreted by IL-13-stimulated synoviocytes may be an important contributor to KOA progression. Furthermore, chondroitin sulfate, a KOA therapeutic agent, suppresses periostin production stimulated by IL-13 [17].

Boiogito (BOT), a traditional herbal medicine known as Kampo medicine in Japan, comprises a dry extract that contains *Sinomenium *stem (5.0 g), *Astragalus* root (5.0 g), *Atractylodes* *lancea *rhizome (3.0 g), jujube (3.0 g), Glycyrrhiza (1.5 g), and ginger (1.0 g). These herbal components are blended and extracted with purified water at 95.1°C for 1 h, and the resulting soluble extract is officially recognized by the Japanese Ministry of Health, Labour and Welfare [18]. According to oriental medicine principles, BOT enhances water metabolism, reduces swelling, and alleviates joint pain, particularly in the knee joint [19]. BOT (Tsumura & Co. (TJ-20; Lot No. 2190020010), Tokyo, Japan) has been prescribed to alleviate swelling and pain associated with KOA. Oral intake of BOT has been found to alleviate gait pain in patients with KOA, and certain preliminary studies have indicated its ability to suppress inflammatory responses in the knee joint [20,21]. Our previous research showed that BOT enhances locomotive function in rats with surgically induced KOA and impedes the progression of the disease, suggesting its potential in preventing KOA progression [22]. However, whether BOT suppresses periostin production in response to IL-13 stimulation in synovial cells remains unclear. Therefore, this study investigated the preventive effect of BOT on periostin secretion in hFLS stimulated by IL-13. Therefore, this study aims to investigate the preventive effect of the traditional herbal medicine BOT on periostin secretion in hFLS stimulated by IL-13, which is implicated in the pathophysiology of KOA. The study also aims to assess the potential of BOT in modulating IL-13-mediated inflammatory pathways, phosphorylation of STAT6, and periostin mRNA expression in hFLS.

## Materials and methods

Synoviocyte Growth (SG) Medium was purchased from Cell Applications, Inc. (San Diego, USA). Recombinant human IL-13 was purchased from R & D Systems, Inc. (Minneapolis, USA) as a preservative-free pure powder. Furthermore, IL-13 was dissolved in phosphate-buffered saline (PBS) with 0.1% bovine serum albumin, sterilized, and stored at −80℃ until use. BOT, purchased from Tsumura Co., Ltd. (Tokyo, Japan), was initially dissolved in PBS containing 0.1% dimethyl sulfoxide at a concentration of 50 mg/mL. Subsequently, the solution was heated at 90°C for 1 h to dissolve the freeze-dried BOT powder as much as possible. Centrifugation was then performed at 25°C for 10 min at 3,000 rpm, followed by sterilization of the supernatant through a 0.2-μm filter. The BOT solution was further dissolved in SG Medium and stored at −80℃ until use.

The hFLS cell line obtained from the synovial tissues in the knee joint of a healthy subject (Cell Applications, Inc., San Diego, USA) was suspended in SG Medium at a concentration of 1 × 10^5^ cells/mL in 3rd to 7th passages. To examine the influence of IL-13 on periostin production by hFLS, 5 × 10^4^ cells (500 µL) were introduced into 24-well culture plates in triplicate and stimulated with various concentrations of IL-13 in a final volume of 1.0 mL. After 24-72 h, the culture supernatants were collected and stored at −80°C until use. To examine the influence of BOT on periostin production from hFLS, 5 × 10^4^ cells (500 µL) were stimulated in triplicate with 20.0 ng/mL IL-13 in the presence of 100-1,000 μg/mL BOT in a final volume of 1.0 mL. After 72 h, the culture supernatants were obtained and stored at −80°C until use. Periostin concentration was measured in duplicate using enzyme-linked immunosorbent assay (ELISA) test kits (EK-074-41, Phoenix Pharmaceuticals, Inc., Burlingame, USA) according to the manufacturer’s recommendations. The minimum detectable level of this kit was 0.027 ng/mL. To prepare cells for examining the influence of BOT on transcription factor signal transducer and activator of transcription factor 6 (STAT6) activation and periostin mRNA (POSTN) expression in hFLS after IL-13 stimulation, 5 × 10^4^ cells (500 µL) were stimulated in triplicate with 20.0 ng/mL IL-13 in the presence of 100-1,000 μg/mL BOT in 1.0 mL for 30 min and 24 h, respectively. In all experiments, BOT was added to the cell cultures 2 h before stimulation.

The influence of BOT on cell viability was examined using the MTT assay. Cells suspended in the SG Medium at a concentration of 1 × 10^5^ cells/mL were introduced into each well of a 48-well culture plate that contained 20 ng/mL of IL-13 and various concentrations of BOT in 1.0 mL in triplicate. After 72 h, 5 mg/mL of MTT solution (100 μL) was added to each well and incubated for 3 h. The MTT solution in each well was removed, and 300 μL of MTT solubilization buffer (0.04 N HCl in isopropanol) was added to dissolve formazan. The lysate was applied to each well of a 96-well culture plate in a volume of 100 µL in duplicate for each sample. The absorbance at 570 nm was measured using a microplate reader (Multiskan GO, Thermo-Fisher Scientific, Waltham, USA).

Periostin mRNA expression in cultured cells was determined using real-time quantitative reverse transcription polymerase chain reaction (qRT-PCR). RNA was extracted from cells 24 h after IL-13 stimulation using the RNeasy Mini Kit (74106, QIAGEN, Hilden, Germany), and cDNA was synthesized using ReverTra Ace qPCR RT Master Mix with gDNA Remover (FSQ-301, TOYOBO, Osaka, Japan) according to the manufacturer’s instructions. PCR was then performed using QuantStudio 3 (Applied Biosystems, Forster City, USA). The PCR mixture comprised 1.0 μL of sample cDNA solution (150 ng/μL), 10.0 μL of SYBR-Green Mastermix (Applied Biosystems), 0.2 μL of sense and antisense primers, and distilled water to give a final volume of 18.5 μL. The reaction was conducted as follows: 2 min at 95℃, followed by 40 cycles of 15 s at 95℃ and 60 s at 60℃ and final elongation for 15 s at 95℃, 60 s at 60℃, and 15 s at 95℃ to obtain the dissociation curve. Glyceraldehyde 3-phosphate dehydrogenase (GAPDH) was amplified as an internal control mRNA. Periostin mRNA levels were calculated using the comparative parameter threshold cycle and normalized against GAPDH. The nucleotide sequences of the primers were as follows: for periostin, 5′-ACAGCTCAGAGTCTTCGTATATCG-3′ (sense) and 5′-CCGTTTCTCCCTTGCTTACTCC-3′ (antisense), and for GAPDH, 5′-TGCACCACCAACTGCT-3′ (sense) and 5′-GGCATGGACTGTGGTC-3′ (antisense).

STAT6 phosphorylation in cultured cells was assessed by detecting the levels of relative phosphorylated STAT6 (pSTAT6) normalized with total STAT6 in hFLS using Western blotting. The cells were homogenized in lysis buffer containing 1% sodium dodecyl sulfate (SDS), 20 mM Tris-HCl (pH 7.4), 5 mM ethylenediaminetetraacetic acid (pH 8.0), 10 mM sodium fluoride, 2 mM sodium orthovanadate, 0.5 mM phenyl arsine oxide, and 1 mM phenylmethylsulfonyl fluoride. The lysate was centrifuged at 15,000 rpm for 30 min at 25℃, and the supernatant was collected. The concentrations of all samples were standardized using the BCA protein assay kit (Thermo-Fisher Scientific). Samples containing an equivalent amount of proteins were subjected to sodium dodecyl sulfate (SDS)-polyacrylamide gel electrophoresis (10% SDS) and then transferred onto polyvinylidene difluoride membranes. Membranes were immersed in 5% (w/v) bovine serum albumin (#011-21271, FUJIFILM Wako Pure Chemical, Richmond, USA) containing Tris-buffered saline buffer and Tween 20 (TBST) (Sigma-Aldrich Japan Co., Tokyo, Japan) for 1 h at room temperature. After washing the membranes with TBST, they were incubated overnight at 4°C with an anti-pSTAT6 antibody (1:1,000, #9361S, Cell Signaling Technology) or anti-STAT6 antibody (1:1,000, #5397S, Cell Signaling Technology, Danvers, USA). The membranes were washed with TBST and incubated with goat antirabbit secondary antibody conjugated with horseradish peroxidase (1:1000, #611-1302, Rockland Immunochemicals, Gilbertsville, USA) for 1 h at room temperature. Chemiluminescence images using a Pierce™ ECL Western blotting substrate (Thermo-Fisher Scientific) were captured using a charge-coupled device camera system (Ez-Capture MG, Atto Co., Tokyo, Japan). The immunoreactivity of each band was measured using the Lane & Spot Analyzer software (Atto Co., Ltd.), and the fold change in the density of pSTAT6 in each sample was normalized to that of total STAT6.

To confirm that transcriptional activity through STAT6 phosphorylation is the major response in intracellular signaling involved in periostin production following IL-13 stimulation, the STAT6 inhibitor, 10-100 nM of AS1517499 (Selleck Chemicals, Houston, USA), was administered in a 30 min prior to IL-13 application. After 72 h, the culture supernatants were obtained and stored at −80°C until use. Periostin concentration was measured in triplicate with ELISA.

Statistical analyses were performed using the JMP Pro (Version 16.0; SAS Institute Inc., Cary, USA), and significance was set at a *P*-value < 0.05. Normality tests were performed for the explanatory variables, and variables showing normal distribution are presented as means ± standard deviations. The Dunnett or Tukey test was performed as a post hoc analysis after analysis of variance to examine significant differences in multigroup comparisons.

## Results

**Figure 1:**
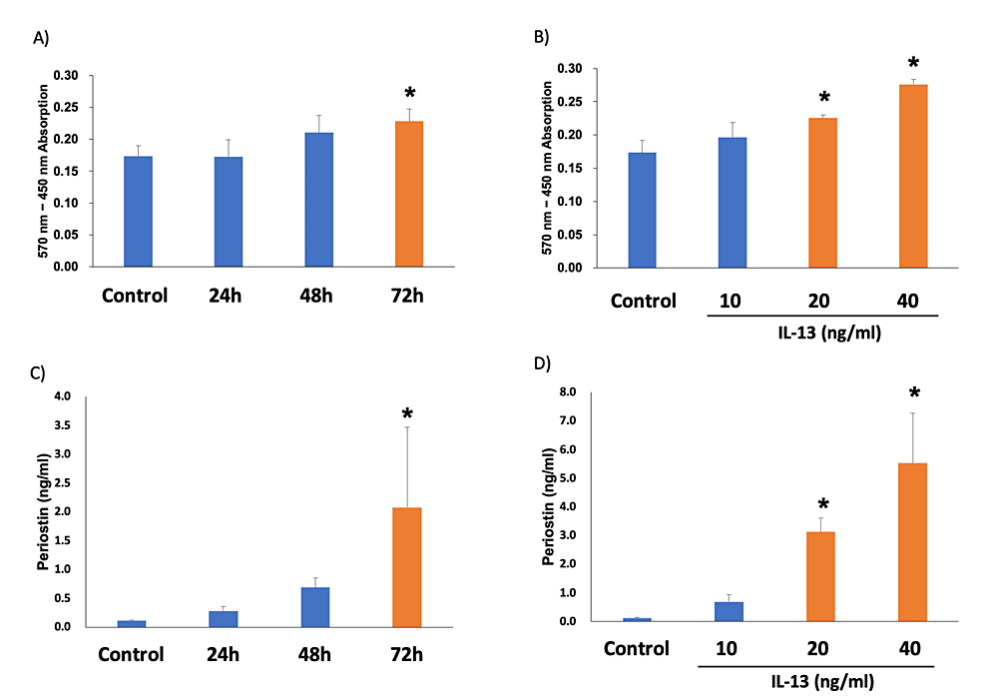
IL-13 stimulation in hFLS. A) MTT assay for the time-response of IL-13 stimulation on hFLS. B) MTT assay for the dose-response of IL-13 stimulation on hFLS. C) ELISA for periostin secretion to assess the time-response of IL-13 stimulation. D) ELISA for periostin secretion to assess the dose-response of IL-13 stimulation.*P < 0.05 *versus *the control group based on ANOVA followed by the Dunnett test. ANOVA, analysis of variance; ELISA, enzyme-linked immunosorbent assay; hFLS, human fibroblast-like synoviocytes; IL-13, interleukin-13; MTT, 3-(4,5-dimethylthiazol-2-yl)-2,5-diphenyl-2H-tetrazolium bromide

Periostin secretion by hFLS was assessed by detecting periostin levels in the hFLS culture medium. The cells were treated with IL-13. Periostin levels were measured using a commercially available MTT assay and ELISA. An IL-13 time-response curve was established using a fixed concentration of 20 ng/mL IL-13 for 24, 48, and 72 h, whereas an IL-13 dose-response curve was generated by treating cells with different concentrations of IL-13 for 72 h. The results of the MTT assay for the time-response of IL-13 stimulation on hFLS were as follows: 0.174 ± 0.016 in the control, 0.173 ± 0.026 at 24 h, 0.210 ± 0.027 at 48 h, and 0.228 ± 0.019 at 72 h (Figure 1A). The results for the MTT assay for the dose-response of IL-13 stimulation on hFLS were as follows: 0.174 ± 0.018 ng/mL in the control, 0.196 ± 0.022 ng/mL in 10 ng/mL, 0.225 ± 0.005 ng/mL in 20 ng/mL, 0.276 ± 0.007 ng/mL in 40 ng/mL (Figure 1B). The ELISA results for periostin secretion to assess the time-response of IL-13 stimulation were as follows: 0.12 ± 0.01 ng/mL in the control, 0.28 ± 0.09 ng/mL at 24 h, 0.69 ± 0.17 ng/mL at 48 h, and 2.08 ± 1.40 ng/mL at 72 h (Figure 1C). The ELISA results for periostin secretion to assess the dose-response of IL-13 stimulation were as follows: 0.13 ± 0.01 ng/mL in the control, 0.69 ± 0.26 ng/mL in 10 ng/mL, 3.12 ± 0.49 ng/mL in 20 ng/mL, and 5.53 ± 1.75 ng/mL in the 40 ng/mL (Figure 1D). These results showed a significant increase in periostin concentrations when IL-13 levels exceeded 20 ng/mL and after ≥72 h. Subsequent experiments with synoviocytes were performed based on these established IL-13 thresholds.

**Figure 2:**
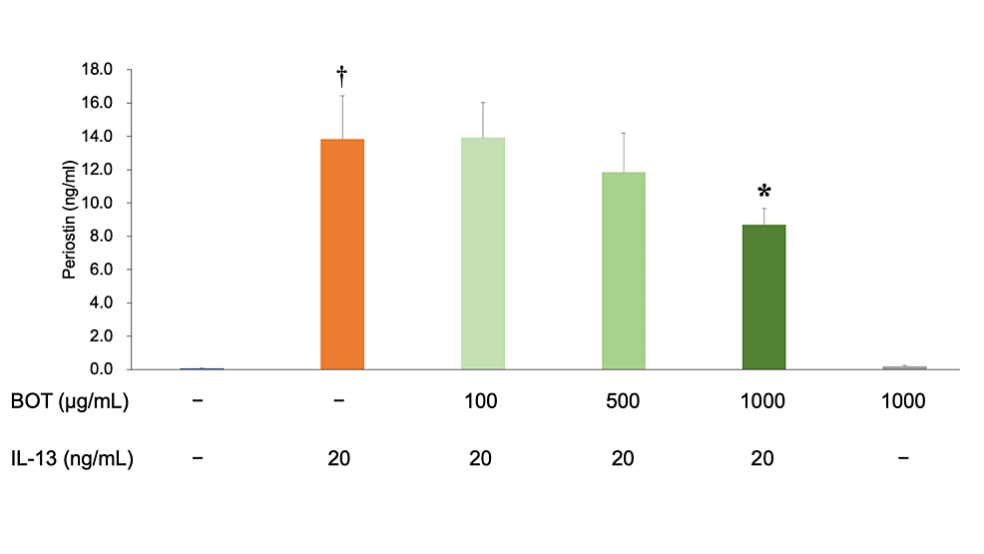
Suppressive effect of BOT on periostin protein secretion as measured by ELISA. Periostin secretion by hFLS was assessed by detecting the periostin levels in the hFLS culture medium. The cells were treated with IL-13 (20 ng/mL for 72 h). BOT was applied 2 h before IL-13 application. Periostin levels were measured using a commercially available ELISA. ^†^*P* < 0.05 *versus* the control group; *P < 0.05 *versus* the IL-13 group based on ANOVA followed by the Tukey test. ANOVA, analysis of variance; BOT, boiogito; IL-13, interleukin-13

Periostin secretion by hFLS was assessed by detecting the periostin levels in the hFLS culture medium. The cells were treated with IL-13 (20 ng/mL for 72 h). BOT was applied 2 h before IL-13 application. Periostin levels were measured using a commercially available ELISA. The results were as follows: 0.09 ± 0.21 ng/mL in the control, 13.82 ± 2.59 ng/mL in IL-13, 13.92 ± 2.11 ng/mL in IL-13 + BOT (100 µg/mL), 11.84 ± 2.36 ng/mL in IL-13 + BOT (500 µg/mL), 8.70 ± 0.98 ng/mL in IL-13 + BOT (1,000 µg/mL), and 0.19 ± 0.05 ng/mL in BOT (1,000 µg/mL) (Figure 2). Administration of 1,000 μg/mL of BOT significantly decreased periostin secretion.

**Figure 3:**
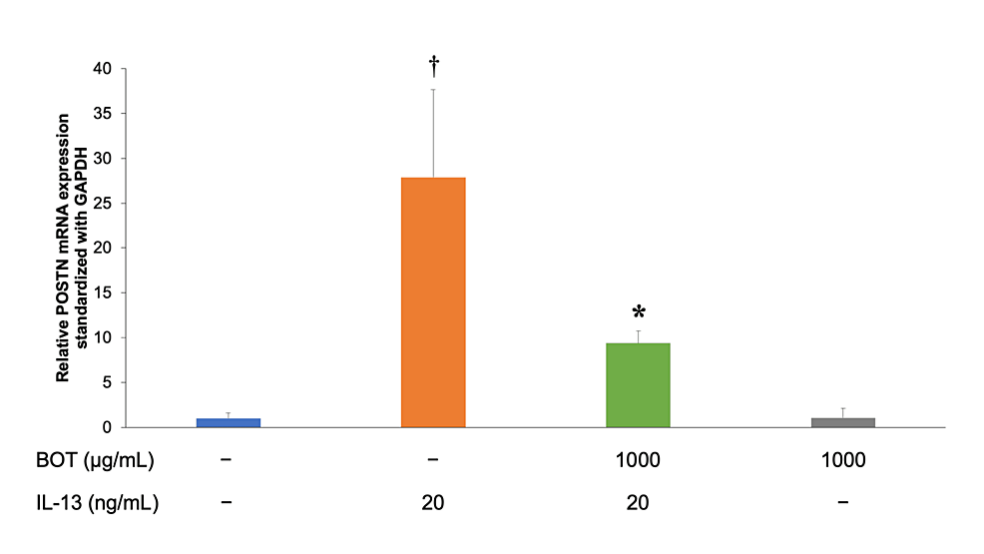
Suppressive effect of BOT on POSTN mRNA expression measured by qRT-PCR. POSTN mRNA expression by hFLS was assessed by detecting periostin mRNA levels in hFLS. The cells were treated with IL-13 (20 ng/mL for 24 h). Next, 1,000 μg/mL of BOT was applied 2 h before IL-13 application. Periostin (POSTN) mRNA expression was measured using qRT-PCR. ^†^*P* < 0.05 *versus* the control group; *P < 0.05 *versus* the IL-13 group based on ANOVA followed by the Tukey test. ANOVA, analysis of variance; BOT, boiogito; GAPDH, glyceraldehyde-3-phosphate dehydrogenase; IL-13, interleukin-13; mRNA, messenger ribosomal nucleotide acid; POSTN, periostin; qRT-PCR, quantitative real-time polymerase chain reaction; hFLS, human fibroblast-like synoviocytes

POSTN mRNA expression by hFLS was assessed by detecting periostin mRNA levels in hFLS. The cells were treated with IL-13 (20 ng/mL for 24 h). Next, 1,000 μg/mL of BOT was applied 2 h before IL-13 application. Periostin (POSTN) mRNA expression was measured using qRT-PCR. The results were as follows: 1 ± 0.62 in the control, 27.91 ± 9.75 in IL-13, 9.39 ± 1.38 in IL-13 + BOT (1,000 µg/mL), and 1.06 ± 1.07 in BOT (1,000 µg/mL) (Figure 3). POSTN mRNA expression significantly decreased after BOT administration in IL-13-stimulated hFLS.

**Figure 4:**
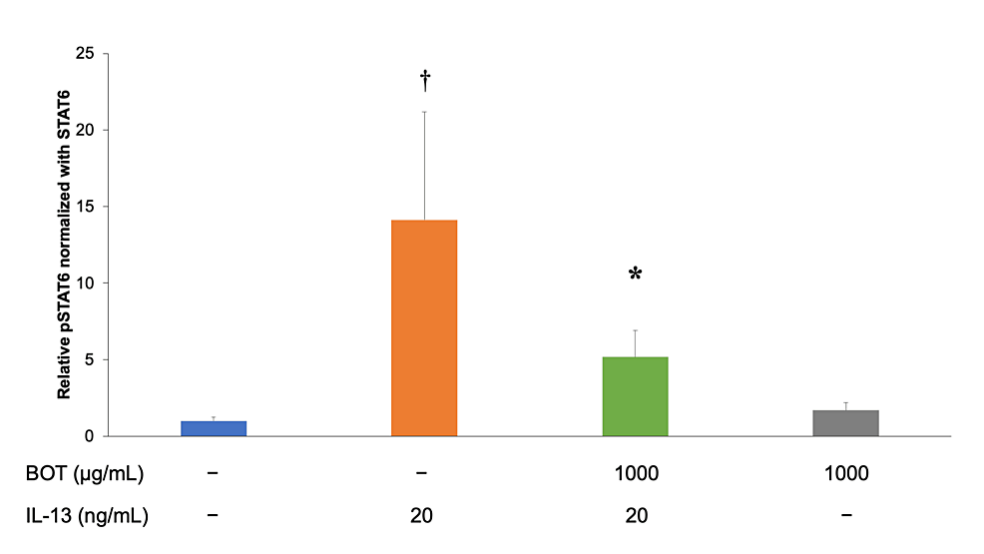
Suppressive effect of BOT on STAT6 phosphorylation measured by Western blotting. STAT6 phosphorylation was assessed by detecting the levels of relative pSTAT6 normalized with STAT6 in hFLS. The cells were treated with IL-13 (20 ng/mL for 30 min). Next, 1,000 μg/mL of BOT was applied 2 h before IL-13 application. Relative pSTAT6 normalized with STAT6 was measured using Western blotting. ^†^*P* < 0.05 *versus* the control group; *P < 0.05 *versus* the IL-13 group based on ANOVA followed by the Tukey test. ANOVA, analysis of variance; BOT, boiogito; IL-13, interleukin-13; pSTAT6, phosphate signal transducer and activator of transcription-6; STAT-6, signal transducer and activator of transcription-6

STAT6 phosphorylation was assessed by detecting the levels of relative pSTAT6 normalized with STAT6 in hFLS. The cells were treated with IL-13 (20 ng/mL for 30 min). Next, 1,000 μg/mL of BOT was applied 2 h before IL-13 application. Relative pSTAT6 normalized with STAT6 was measured using Western blotting. The results were as follows: 1.0 ± 0.2 in the control, 14.1 ± 7.1 in IL-13, 5.2 ± 1.7 in IL-13 + BOT (1,000 µg/mL), and 1.7 ± 0.5 in BOT (1,000 µg/mL)(Figure 4). Relative pSTAT6 normalized with STAT6 significantly decreased after BOT administration in IL-13-stimulated hFLS.

**Figure 5:**
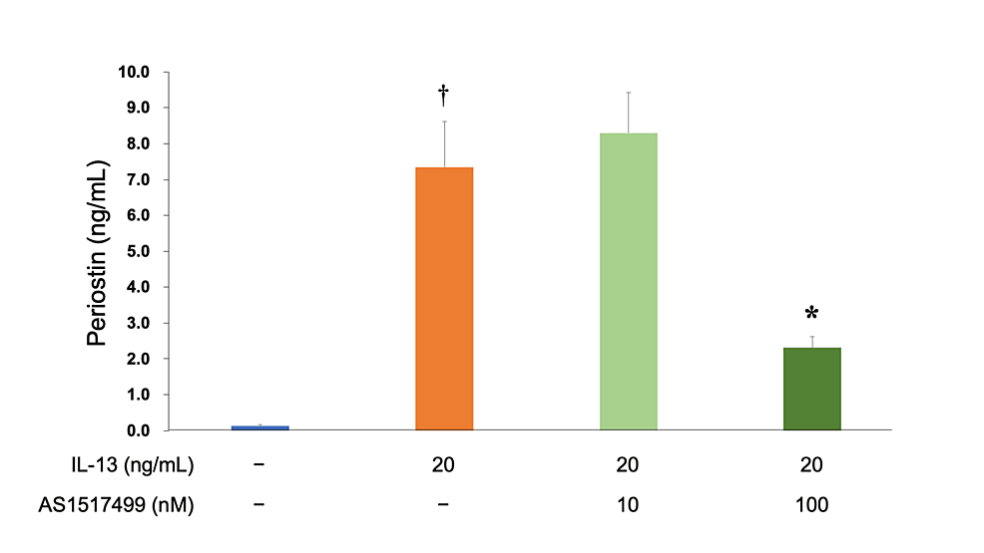
Suppressive activity of AS1517499 for protein secretion measured by ELISA. ^†^*P* < 0.05 *versus *the control group; *P < 0.05 *versus* the IL-13 group based on ANOVA followed by the Tukey test. ANOVA, analysis of variance; IL-13, interleukin-13; ELISA, enzyme-linked immunosorbent assay

Additionally, pre-administration of AS1517499 before IL-13 stimulation to hFLS significantly inhibited periostin secretion. Periostin levels were assessed with ELISA. The results were as follows: 0.13 ± 0.04 ng/mL in the control, 7.35 ± 1.28 ng/mL in IL-13, 8.30 ± 1.13 ng/mL in IL-13 + AS1517499 (10 nM), and 2.31± 0.31 ng/mL in IL-13 + AS1517499 (100 nM). Administration of 100 nM of AS1517499 significantly decreased periostin secretion (Figure 5).

## Discussion

This study explored the preventive impact of BOT on periostin secretion in hFLS stimulated by IL-13 in vitro. Periostin expression was induced by 20 ng/mL of IL-13 and significantly increased from 72 h after stimulation. Furthermore, 1,000 μg/mL of BOT 2 h before IL-13 stimulation significantly reduced periostin expression. Pre-administration of BOT prevented the IL-13-dependent intracellular signaling cascade for periostin secretion by inhibiting STAT6 phosphorylation.

Periostin, categorized within the fasciclin family, is present in connective tissues, such as tendons, ligaments, periosteum, and heart valves. Periostin plays a vital role in tissue repair; however, excessive or prolonged periostin expression can intensify the development of tumors, bronchial asthma, atopic dermatitis, polycystic kidney disease, and other fibrotic conditions. Studies have shown the effect of periostin in KOA and indicated that periostin is upregulated in OA synovial fibroblasts [16]. Furthermore, periostin levels in the synovial fluid increased with KOA progression and may be a biomarker for estimating the severity of synovitis [15]. Moreover, a previous study showed that knockout mice lacking the periostin gene exhibit suppressed KOA progression [23]. The results of our current study revealed that stimulation with IL-13 leads to the production of periostin from hFLS. The amount produced increased over 100 times compared with the control (Figure 2).

In this study, IL-13 was used as a chemical mediator to stimulate hFLS. IL-13 serves as a hallmark cytokine of type 2 inflammation generated by TH2 cells, follicular helper T cells, group 2 innate lymphoid cells, eosinophils, mast cells, and basophils. Its significance extends beyond allergic conditions, such as asthma, allergic rhinitis, and atopic dermatitis, because it also contributes to the pathogenesis of various inflammatory disorders, including chronic obstructive pulmonary disease, certain cancers, inflammatory bowel diseases, autoimmune conditions, and pulmonary fibrosis [24]. Furthermore, IL-13 is reported to be increased in the synovial fluid of patients with rheumatoid arthritis, which is an autoimmune disease targeting the synovial tissue [25]. IL-13, along with IL-4 and IL-10, have anti-inflammatory effects and can regulate inflammatory responses and suppress the development of OA [26]. However, interpreting the results of this study, IL-13 stimulation on hFLS increased periostin production, suggesting that IL-13 contributes to synovitis. The critical issue here is that during KOA progression, periostin is believed to be persistently secreted in large quantities. From this study, whether hFLS stimulated by IL-13 undergo phenotypic transformation to continuously produce periostin remains unclear; therefore, further research is required.

STAT6 plays a pivotal role in the signaling pathway of IL-13. Upon binding of IL-13 to its receptor, Janus kinases are activated, leading to STAT6 phosphorylation. Phosphorylated STAT6 molecules form dimers and translocate to the nucleus. In the nucleus, pSTAT6 dimers bind to specific DNA sequences known as response elements. By binding to specific DNA sequences, STAT6 regulates the transcriptional activities of genes targeted by IL-13. This can lead to the expression of genes involved in immune responses, inflammation, and other biological processes influenced by IL-13 signaling. Overall, STAT6 serves as a crucial transcription factor in the IL-13 signaling pathway, mediating cellular responses to IL-13 by regulating the expression of target genes [24]. The results showed that periostin production induced by IL-13 stimulation in hFLS was almost completely inhibited. It was revealed that periostin production in hFLS stimulated by IL-13 largely depends on intracellular signaling mediated by STAT6.

Our study showed that administering BOT significantly suppressed periostin production. Moreover, it became evident that the suppressive effect occurred before STAT6 phosphorylation in the intracellular signal cascade via the IL-13 receptor. A previous study reported that sinomenine treatment improved the arthritic score and hind paw swelling in rat models of adjuvant- and collagen-induced arthritis [27]. Another study showed that astragalus root exhibits anti-inflammatory effects by reducing the production of cytokines in various animal models, including autoimmune myocarditis in rats [28]. However, it remains unclear which herbal components of BOT contributed to this effect; therefore, further research is required to clarify this issue.

The present study has several limitations. The first limitation of this study is that, although there are numerous chemical mediators involved in the onset and progression of KOA of the knee, the experiments were focused solely on the action of IL-13. While IL-13 is recognized as an anti-inflammatory cytokine [26], IL-13 suggests its involvement in sensitization related to type 2 innate immunity in allergic diseases such as bronchial asthma [24], implying its essential role may vary depending on the disease. However, this study revealed that the action on hFLS led to increased production of periostin, suggesting its potential involvement in the onset and progression of KOA. The second limitation is the lack of clarity on whether the increased production of periostin directly contributes to the progression of KOA. Although studies with periostin-related gene knockout animals have shown inhibition of KOA progression, it remains unclear whether the levels of periostin measured in this study are sufficient to induce the progression of KOA. Future research, including animal experiments, is needed to clarify whether intra-articular injection of IL-13 or periostin contributes to the development and progression of KOA.

Although this study elucidates the suppressive activity of BOT on IL-13-induced periostin secretion in hFLS through STAT6 phosphorylation, there are some limitations. Firstly, it remains unclear whether 20 ng/mL of IL-13 is sufficient to trigger synovitis in the articular joint. Therefore, it is necessary to investigate the IL-13 levels in the synovial fluid from KOA patients and to conduct *in vivo* animal studies to determine if intraarticular administration of the same level of IL-13 induces arthritis. Since this is also relevant for periostin, addressing the research described above is important. Secondly, since BOT is administered orally, it is unknown how much of the compound is absorbed and transferred to the bloodstream once it is digested. However, Kimura et al. [29] showed that 1% of BOT mixed with the rats' chow inhibited KOA progression in a rat KOA model, suggesting that a relatively small amount of BOT with oral administration can sufficiently exert its effects. Therefore, 1,000 µg/mL of BOT might be appropriate.

## Conclusions

This study demonstrates that IL-13 stimulation induces periostin production in hFLS, suggesting that IL-13 plays an important role in synovitis associated with KOA. Furthermore, BOT administration significantly suppresses periostin secretion in IL-13-stimulated hFLS, potentially by inhibiting STAT6 phosphorylation. These findings suggest that BOT has a potential therapeutic effect on attenuating the inflammatory response associated with KOA by targeting periostin production. Further investigation into the specific herbal components of BOT responsible for this effect is warranted to fully elucidate its mechanism of action. Overall, these results provide insights into the potential of BOT as a preventive therapeutic option for KOA, offering hope for alleviating the burden of its debilitating condition on patients’ QOL.
